# Detection of Respiratory Viruses Other Than SARS‐CoV‐2 in a Large Hospital Laboratory in Rome, Italy, During the Seasons 2016–2017 to 2022–2023

**DOI:** 10.1111/irv.70079

**Published:** 2025-02-20

**Authors:** Flora Marzia Liotti, Simona Marchetti, Sara D'Onghia, Maurizio Sanguinetti, Rosaria Santangelo, Brunella Posteraro

**Affiliations:** ^1^ Dipartimento di Scienze di Laboratorio ed Ematologiche Fondazione Policlinico Universitario A. Gemelli IRCCS Rome Italy; ^2^ Dipartimento di Scienze Biotecnologiche di Base, Cliniche Intensivologiche e Perioperatorie Università Cattolica del Sacro Cuore Rome Italy; ^3^ Dipartimento di Scienze Mediche e Chirurgiche Fondazione Policlinico Universitario A. Gemelli IRCCS Rome Italy

**Keywords:** influenza, molecular detection, respiratory syncytial virus, respiratory viruses, SARS‐CoV‐2

## Abstract

**Background and Objective:**

Respiratory viruses are major contributors to morbidity and mortality worldwide, with their circulation influenced by seasonal patterns and pandemic‐related interventions. This study analyzed detection trends of non‐SARS‐CoV‐2 respiratory viruses in a large Italian hospital over a 7‐year period, focusing on variations across COVID‐19–related periods and patient age groups.

**Methods:**

We retrospectively analyzed multiplex PCR‐based laboratory results of 8836 nasopharyngeal samples collected between September 2016 and August 2023. Viral detection rates were stratified by season, COVID‐19–related periods (pre‐pandemic, pandemic, post‐pandemic), and age groups.

**Results:**

Of the 8836 nasopharyngeal samples analyzed, 2795 (31.6%) tested positive for at least one respiratory virus. Rhinovirus/enterovirus (RV/EV) was the most frequently detected virus (37.6%), followed by influenza A virus (IAV, 17.9%) and respiratory syncytial virus (RSV, 17.2%). The 2020–2021 season had the lowest positivity rate (*p* < 0.001), with marked declines in IAV and RSV detections, likely because of COVID‐19 mitigation measures. Conversely, detections of RV/EV and human coronaviruses increased. Postpandemic data suggested a return to prepandemic patterns, though overall positivity rates remained altered. Age‐stratified analysis revealed RSV predominance in infants (*p* < 0.001), emphasizing its clinical relevance in pediatric populations.

**Conclusions:**

Our findings highlight the dynamic nature of respiratory virus epidemiology and the persistent impact of the COVID‐19 pandemic on viral circulation. Continuous surveillance and adaptive public health strategies are essential for managing future outbreaks and mitigating the burden of respiratory viral infections.

## 1 Introduction

Respiratory viruses, including influenza viruses, respiratory syncytial virus (RSV), and human coronaviruses, have long been recognized as leading causes of morbidity and mortality worldwide, particularly among vulnerable populations such as young children, the elderly, and immunocompromised individuals [1, 2, 3]. These infections manifest with a broad clinical spectrum, ranging from mild upper respiratory tract illnesses (e.g., the common cold) to severe lower respiratory tract diseases, such as pneumonia, bronchiolitis, and acute respiratory distress syndrome [4].

More recently, severe acute respiratory syndrome coronavirus 2 (SARS‐CoV‐2), the causative agent of 2019 coronavirus disease (COVID‐19), has emerged as a significant respiratory pathogen, profoundly impacting global public health. Beyond the direct consequences of COVID‐19, the pandemic altered the epidemiology of other respiratory viruses [5, 6]. Stringent infection control measures, including social distancing, masking, and lockdowns, led to an unprecedented decline in the circulation of certain viruses, particularly influenza, during the early phases of the pandemic. However, as these measures were gradually lifted, respiratory viruses such as RSV and influenza re‐emerged with altered incidence, seasonality, and age‐specific prevalence compared with prepandemic patterns [7, 8].

Despite these observations, comprehensive studies investigating long‐term trends of non‐SARS‐CoV‐2 respiratory viruses across different COVID‐19–related periods remain limited. Understanding how these viruses have evolved in response to pandemic‐related interventions is critical to informing surveillance strategies and public health measures.

In this study, we analyze detection trends of RSV, influenza A virus (IAV), influenza B virus (IBV), and other non‐SARS‐CoV‐2 respiratory viruses over a 7‐year period (September 2016–August 2023) using data from the clinical microbiology laboratory of a large hospital in Rome, Italy. Additionally, we assess differences in viral detection across the prepandemic, pandemic, and postpandemic periods, providing insights into the broader epidemiological impact of the COVID‐19 pandemic on respiratory virus circulation.

## 2 Methods

This retrospective study included the analysis of multiplex real‐time polymerase chain reaction (PCR) testing results from upper respiratory tract (i.e., nasopharyngeal) samples, along with the corresponding clinical and demographic data of the patients from whom these samples were derived. Samples were collected between September 1, 2016, and December 31, 2023, from patients presenting to the emergency department or hospitalized within the Fondazione Policlinico Universitario A. Gemelli IRCCS, a tertiary care hospital in Rome, Italy. Patients were suspected of viral infections based on fever and/or respiratory symptoms, such as rhinorrhea, cough, sore throat, or shortness of breath. Samples were processed in the clinical microbiology laboratory of Fondazione Policlinico Universitario A. Gemelli IRCCS, which plays a key role in national and regional networks for the surveillance of respiratory viral infections. To minimize duplication, samples collected within short time intervals from the same patient for monitoring purposes were excluded from the analysis.

Nasopharyngeal swabs were collected by trained healthcare personnel using flocked swabs and placed in Universal Transport Medium (UTM; Copan Diagnostics), which is designed to support sample collection and preservation during transport. Samples were promptly delivered to the clinical microbiology laboratory under controlled conditions and processed within hours of collection to ensure sample integrity.

During the study period, detection of respiratory viruses was performed using commercially available multiplex PCR assays such as Allplex Respiratory Panel 1/2/3 (Seegene), ePlex Respiratory Pathogen Panel 1 (GenMark Diagnostics), BioFire FilmArray Respiratory 2.1plus Panel (bioMérieux), or QIAstat‐Dx Respiratory SARS‐CoV‐2 Panel (Qiagen). For the Allplex assay, nucleic acids were extracted using the Hamilton STARlet platform prior to PCR, while the ePlex, FilmArray, and QIAstat‐Dx assays employed integrated sample‐to‐answer workflows, automating nucleic acid extraction, amplification, and detection within the respective cartridges. These assays covered the following viral targets: adenovirus (AdV), human coronavirus (hCoV), IAV, IBV, human metapneumovirus (hMPV), human parainfluenza virus (hPIV), RSV, and rhinovirus/enterovirus (RV/EV). All assays were performed according to the manufacturers' instructions, ensuring standardized procedures throughout the study period. It is important to note that while samples collected during the study period were likely processed for SARS‐CoV‐2 testing, such results were generated separately and independently from those for other respiratory viruses included in this analysis. Consequently, it was not possible to associate SARS‐CoV‐2 assay results with the results of multiplex PCR assays targeting other respiratory viruses.

Patient demographic and clinical data (e.g., age, sex, patient ward, and date of sample collection) were retrieved retrospectively from laboratory databases. Results were analyzed both overall and stratified by annual seasons, defined as running from September to August. Additionally, results were categorized into three COVID‐19–related periods: pre‐pandemic (January 2017 to February 2020), pandemic (March 2020 to April 2022), and post‐pandemic (May 2022 to December 2023). Positivity rates were calculated as the number of viruses detected divided by the total number of samples tested.

Statistical analysis was performed using SPSS Statistics version 24.0 (IBM Corp.) and GraphPad Prism version 10.2.3 (GraphPad Software). Data were presented as numbers with proportions for categorical variables or as medians with interquartile range (IQR) values for continuous variables, as appropriate. Differences in positivity rates or the distribution of results between study groups (e.g., age groups) were assessed using the chi‐square test, with a *p* value of < 0.05 considered statistically significant.

## 3 Results

A total of 8836 nasopharyngeal samples were collected and tested for respiratory viruses between September 2016 and August 2023. Of these, 4384 samples were obtained from female patients and 4452 from male patients, with a median age of 54 years (IQR, 23–72). Among the 2795 positive samples (31.6%), 1359 were from female patients and 1436 from male patients, with a median age of 17 years (IQR, 1–58).

**FIGURE 1:**
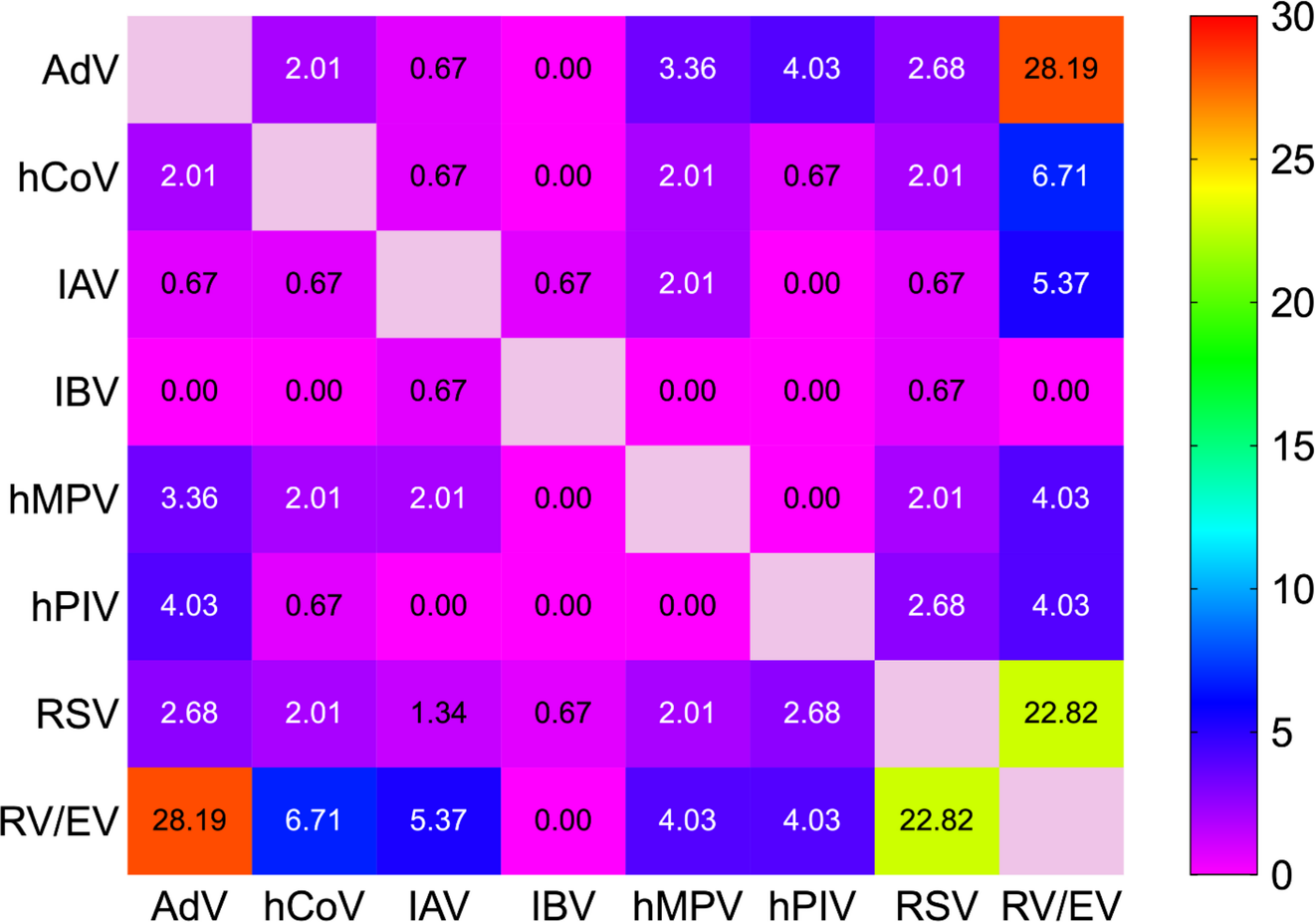
Viral co‐detection rates by PCR for nasopharyngeal samples collected during the study period (September 2016 to August 2023). The percentage values shown are calculated by dividing the number of positive samples for each virus combination by the total number of positive samples for two viral targets (*n* = 149). AdV, adenovirus; hCoV, human coronavirus; IAV, influenza A virus; IBV, influenza B virus; hMPV, human metapneumovirus; hPIV, human parainfluenza virus; RSV, respiratory syncytial virus; RV/EV, rhinovirus/enterovirus.

Co‐detections were observed in 159 samples (5.7% of positive cases), with most involving two viruses (*n* = 149), as shown in Figure 1. RV/EV was the most frequently detected virus, accounting for 1052 cases (37.6% of positive samples), followed by IAV (17.9%), RSV (17.2%), and AdV (10.4%). Less frequently detected viruses included hPIV (7.5%), hCoV (5.6%), hMPV (5.1%), and IBV (4.9%).

**FIGURE 2:**
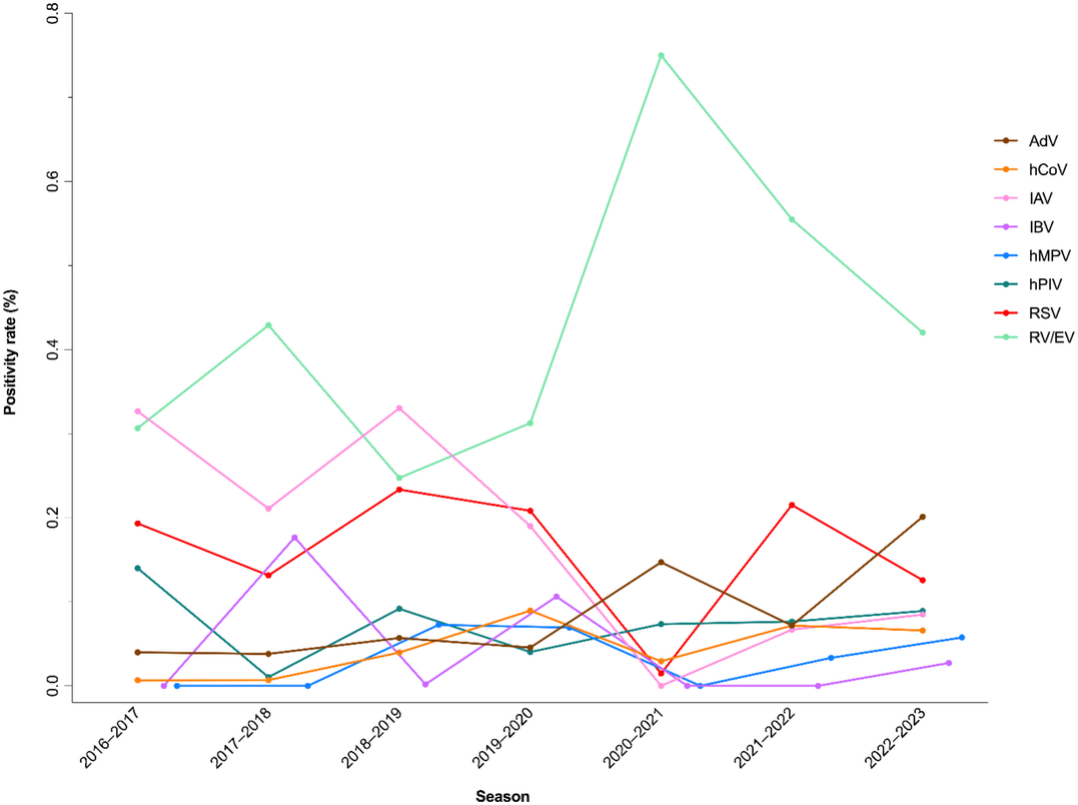
Respiratory virus positivity rates by season for nasopharyngeal samples tested by PCR during the study period (September 2016 to August 2023). For each virus, rates were calculated by dividing the number of positive samples by the number of samples tested in each season (September to August). AdV, adenovirus; hCoV, human coronavirus; IAV, influenza A virus; IBV, influenza B virus; hMPV, human metapneumovirus; hPIV, human parainfluenza virus; RSV, respiratory syncytial virus; RV/EV, rhinovirus/enterovirus.

**FIGURE 3:**
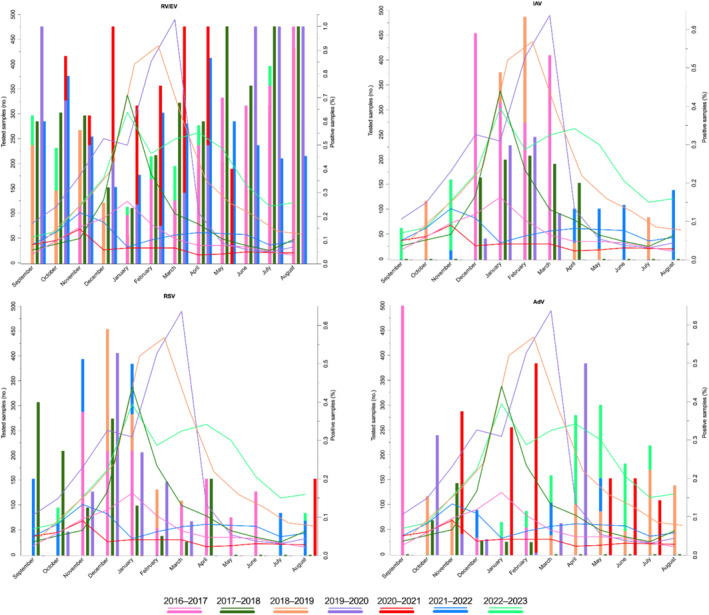
Seasonal trends of viruses most frequently detected by PCR in nasopharyngeal samples from September 2016 to August 2023. For each virus (RV/EV, IAV, RSV, or AdV), bars represent the percentages of positive samples and lines the total number of samples tested. Each annual season, depicted as pink (2016–2017), dark green (2017–2018), orange (2018–2019), violet (2019–2020), red (2020–2021), blue (2021–2022), or light green (2022–2023), is shown from September to August. AdV, adenovirus; IAV, influenza A virus; RSV, respiratory syncytial virus; RV/EV, rhinovirus/enterovirus.

**FIGURE 4:**
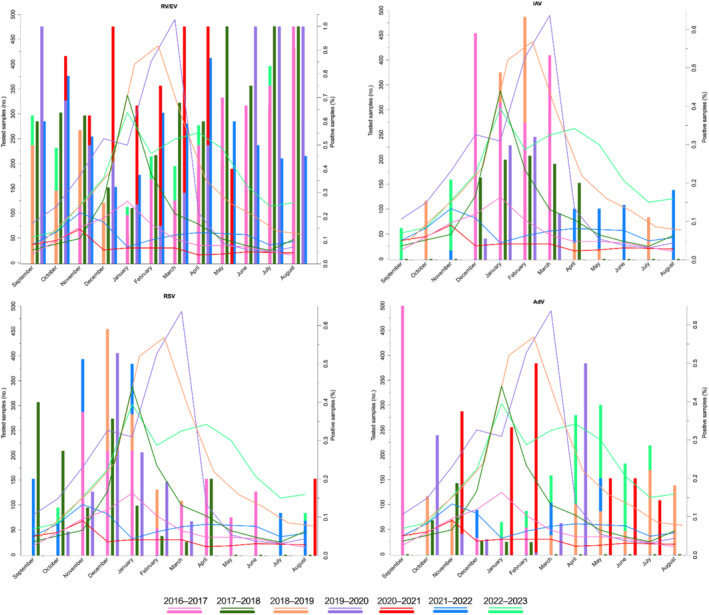
Seasonal trends of viruses less frequently detected by PCR in nasopharyngeal samples from September 2016 to August 2023. For each virus (hPIV, hCoV, hMPV, or IBV), bars represent the percentages of positive samples and lines the total number of samples tested. Each annual season, depicted as pink (2016–2017), dark green (2017–2018), orange (2018–2019), violet (2019–2020), red (2020–2021), blue (2021–2022), or light green (2022–2023), is shown from September to August. hCoV, human coronavirus; IBV, influenza B virus; hMPV, human metapneumovirus; hPIV, human parainfluenza virus.

The 2020–2021 season showed the lowest positivity rate (*p* < 0.001), despite relatively high detection rates for RV/EV (51/68) and AdV (10/68). Overall seasonal detection trends, summarized in Table 1 and illustrated in Figure 2, indicate that positivity rates were highest in the 2018–2019 and 2022–2023 seasons, driven predominantly by RV/EV (143/578 and 401/954, respectively), IAV (191/578 and 81/954, respectively), and RSV (135/578 and 120/954, respectively). In contrast, the 2020–2021 season exhibited the lowest overall positivity rate, likely influenced by public health measures during the COVID‐19 pandemic. When stratified by individual viruses, IAV and IBV were mostly detected in winter months, while hPIV showed higher detection rates during nonwinter months, as illustrated in Figures 3 and 4.

**TABLE 1:** Viral detection results obtained for nasopharyngeal samples collected during the study period (September 2016 to August 2023).

Annual season	Results for viral targets included in multiplex PCR assays used for sample testing								
	All targets	AdV	hCoV	IAV	IBV	hMPV	hPIV	RSV	RV/EV
	No. of samples tested positive/no. of samples tested in total (%)	No. (%) of samples tested positive	No. (%) of samples tested positive	No. (%) of samples tested positive	No. (%) of samples tested positive	No. (%) of samples tested positive	No. (%) of samples tested positive	No. (%) of samples tested positive	No. (%) of samples tested positive
2016–2017	150/614 (24.4)	6 (4.0)	1 (0.7)	49 (32.7)	0 (0.0)	0 (0.0)	21 (14.0)	29 (19.3)	46 (30.7)
2017–2018	289/1089 (26.5)	11 (3.8)	2 (0.7)	61 (21.1)	51 (17.6)	0 (0.0)	3 (1.0)	38 (13.1)	124 (42.9)
2018–2019	578/2067 (28.0)	33 (5.7)	23 (4.0)	191 (33.0)	1 (0.2)	42 (7.3)	53 (9.2)	135 (23.4)	143 (24.7)
2019–2020	547/1973 (27.7)	25 (4.6)	49 (9.0)	105 (19.1)	58 (0.2)	38 (6.9)	22 (4.0)	114 (20.8)	171 (31.3)
2020–2021	68/363 (18.7)	10 (14.7)	2 (2.9)	0 (0.0)	0 (0.0)	0 (0.0)	5 (7.4)	1 (1.5)	51 (75.0)
2021–2022	209/672 (31.1)	15 (7.2)	15 (7.2)	14 (6.7)	0 (0.0)	7 (3.3)	16 (7.7)	45 (21.5)	116 (55.5)
2022–2023	954/2058 (46.4)	192 (20.1)	63 (6.6)	81 (8.5)	26 (2.7)	55 (5.8)	85 (8.9)	120 (12.6)	401 (42.0)
Total	2795/8836 (31.6)	292 (10.4)	155 (5.6)	500 (17.9)	136 (4.9)	142 (5.1)	205 (7.5)	482 (17.2)	1052 (37.6)

*Note:* Results are stratified by annual seasons, each starting in September and ending in August. The total number of positive samples for at least one target (*n* = 2795) included samples with only one target detected (*n* = 2636), samples with two targets detected (*n* = 149), and samples with three targets detected (*n* = 10), accounting for 2964 targets detected in total. Consistently, the sum of the percentages of positive samples for individual viruses in each annual season or in total exceed 100%.

Abbreviations: AdV, adenovirus; hCoV, human coronavirus; IAV, influenza A virus; IBV, influenza B virus; hMPV, human metapneumovirus; hPIV, human parainfluenza virus; RSV, respiratory syncytial virus; RV/EV, rhinovirus/enterovirus.

**FIGURE 5:**
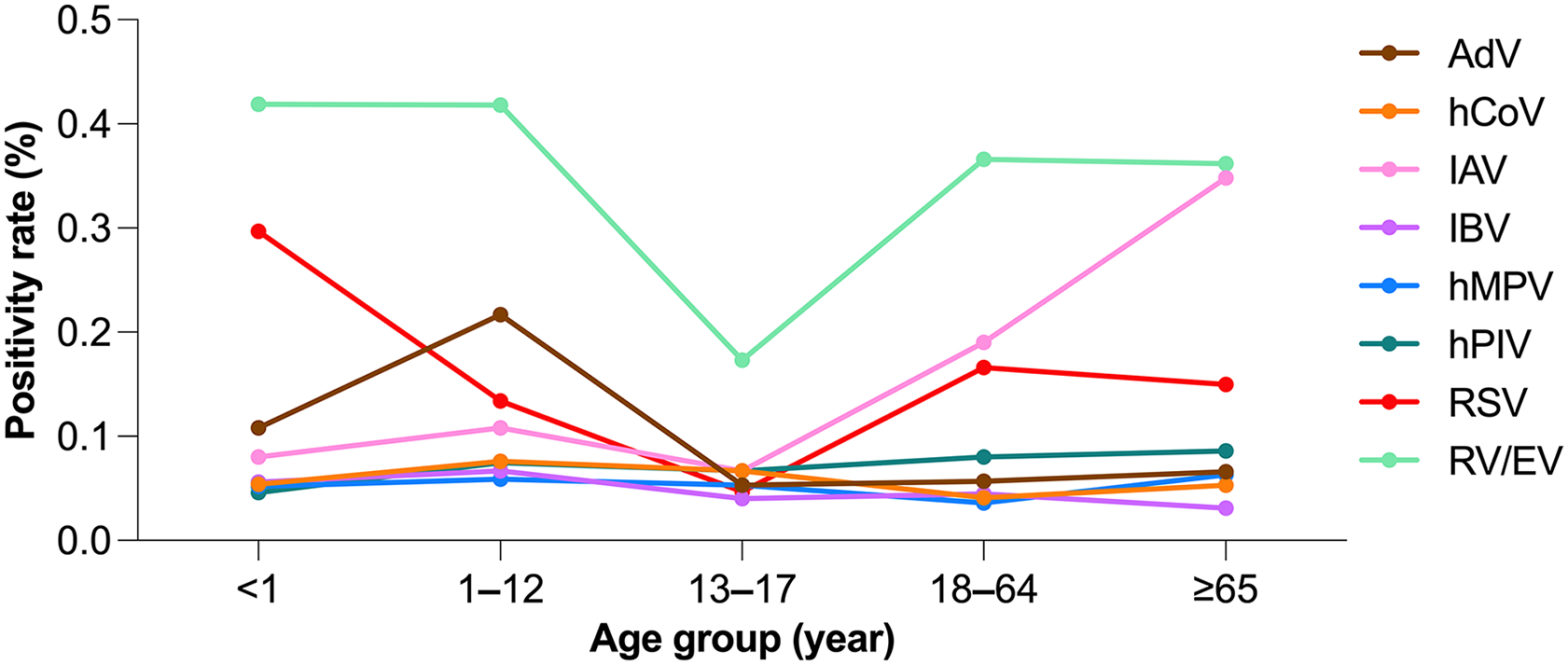
Viral detection results by PCR for nasopharyngeal samples stratified by patient age groups. Samples were collected from September 2016 to August 2023. Age groups included infants (< 1 year), children (1–12 years), adolescents (13–17 years), adults (18–64 years), and older adults (≥ 65 years). AdV, adenovirus; hCoV, human coronavirus; IAV, influenza A virus; IBV, influenza B virus; hMPV, human metapneumovirus; hPIV, human parainfluenza virus; RSV, respiratory syncytial virus; RV/EV, rhinovirus/enterovirus.

Among age‐stratified groups (Figure 5), adolescents (13–17 years) had the lowest number of positive samples for any virus (*p* < 0.001).

**FIGURE 6:**
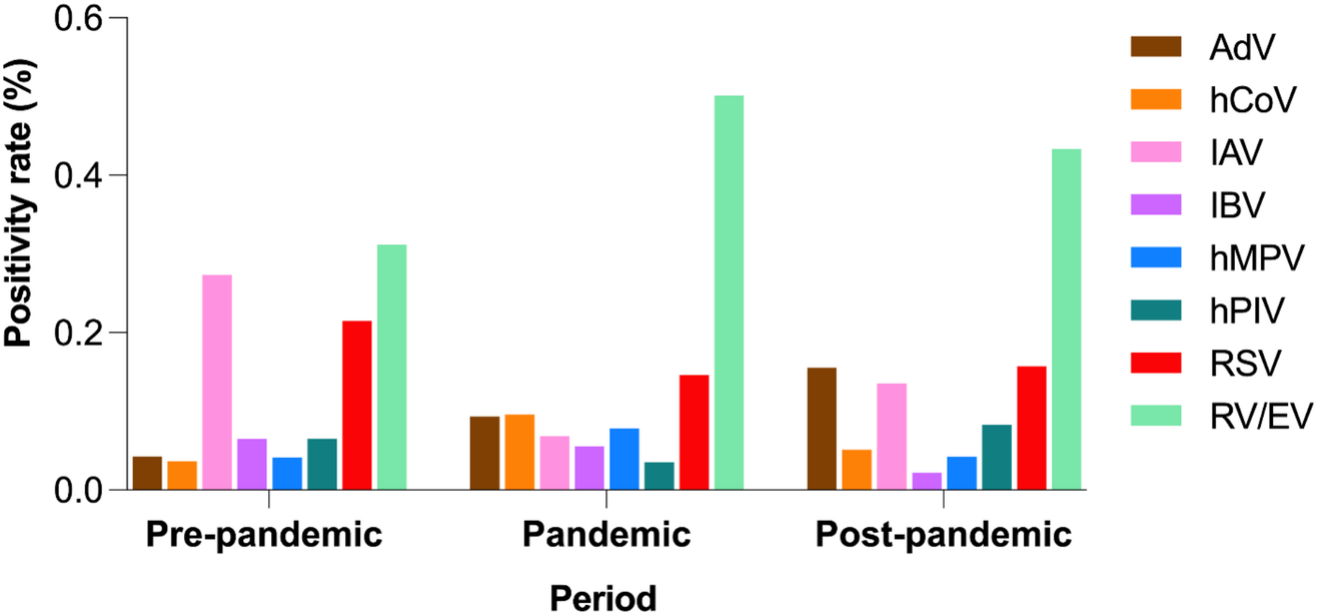
Viral detection results by PCR for nasopharyngeal samples during the prepandemic (January 2017 to February 2020), pandemic (March 2020 to April 2022), and postpandemic (May 2022 to December 2023) periods. AdV, adenovirus; hCoV, human coronavirus; IAV, influenza A virus; IBV, influenza B virus; hMPV, human metapneumovirus; hPIV, human parainfluenza virus; RSV, respiratory syncytial virus; RV/EV, rhinovirus/enterovirus.

Statistically significant differences in positivity rates were observed across the three COVID‐19–related periods (pre‐pandemic, pandemic, and post‐pandemic), as summarized in Table 2 and illustrated in Figure 6.

**TABLE 2:** Comparison of viral detection results for nasopharyngeal samples collected in prepandemic, pandemic, or postpandemic periods of COVID‐19.

Virus detected	Type of period	No. (percentage) of positive results	Percentage change in positivity	*p*	Percentage change in positivity	*p*
Adenovirus (AdV)	Pre‐pandemic	58 (4.3)	Reference			
	Pandemic	37 (9.3)	+5.1	< 0.001	Reference	
	Post‐pandemic	202 (15.5)			+6.2	0.002
Human coronavirus (hCoV)	Pre‐pandemic	50 (3.7)	Reference			
	Pandemic	38 (9.6)	+5.9	< 0.001	Reference	
	Post‐pandemic	67 (5.2)			−4.4	0.002
Influenza A virus (IAV)	Pre‐pandemic	373 (27.3)	Reference			
	Pandemic	27 (6.8)	−20.5	< 0.001	Reference	
	Post‐pandemic	176 (13.5)			+6.7	< 0.001
Influenza B virus (IBV)	Pre‐pandemic	88 (6.5)	Reference			
	Pandemic	22 (5.5)	−0.9	0.588	Reference	
	Post‐pandemic	28 (2.2)			−3.4	< 0.001
Human metapneumovirus (hMPV)	Pre‐pandemic	56 (4.1)	Reference			
	Pandemic	31 (7.8)	+3.7	0.004	Reference	
	Post‐pandemic	55 (4.2)			−3.6	0.007
Human parainfluenza virus (hPIV)	Pre‐pandemic	89 (6.5)	Reference			
	Pandemic	14 (3.5)	−3.0	0.034	Reference	
	Post‐pandemic	108 (8.3)			+4.8	0.002
Respiratory syncytial virus (RSV)	Pre‐pandemic	293 (21.5)	Reference			
	Pandemic	58 (14.6)	−6.9	0.003	Reference	
	Post‐pandemic	205 (15.8)			+1.1	0.064
Rhinovirus/enterovirus (RV/EV)	Pre‐pandemic	425 (31.2)	Reference			
	Pandemic	199 (50.1)	+19.0	< 0.001	Reference	
	Post‐pandemic	564 (43.3)			−6.8	0.020

*Note:* Three COVID‐19–related periods were identified during the study: prepandemic period, starting in January 2017 and ending in February 2020; pandemic period, starting in March 2020 and ending in April 2022; and post‐pandemic period, starting in May 2022 and ending in December 2023.

## 4 Discussion

This study examines respiratory virus detection trends over a 7‐year period (2016–2023) in a tertiary care hospital in Rome, Italy. Our findings indicate notable fluctuations in viral circulation across seasons and different COVID‐19–related periods. Whereas some viruses declined during the pandemic and resurged post‐pandemic, others showed distinct patterns of persistence or fluctuation. These findings underscore the need for continuous monitoring of respiratory virus circulation to better understand the lasting impact of pandemic‐related interventions and changes in population immunity on viral epidemiology.

During the pandemic period, we observed a significant decrease in the detection of IAV, RSV, and hPIV, likely because of the implementation of public health measures such as social distancing, mask‐wearing, and lockdowns. Italy, like other European countries, adopted various strategies to limit the spread of COVID‐19 [9]. Despite these measures, RV/EV, hCoV, and hMPV showed increased detection rates, which may be attributed to their higher transmission potential and resilience to environmental changes [10]. In the postpandemic period, the resurgence of IAV and other respiratory viruses suggested a return to prepandemic circulation patterns, although overall positivity rates remained altered compared with the prepandemic period. Only AdV and IBV showed distinct fluctuations—AdV increasing and IBV decreasing—between COVID‐19–related periods, seemingly independent of SARS‐CoV‐2 circulation. This suggests that while some viruses rapidly resumed their typical seasonal patterns [10], others exhibited a delayed recovery, possibly because of the lingering effects of the pandemic on population immunity and virus transmission dynamics [11].

Our age‐stratified analysis confirmed that RSV was most frequently detected in infants, consistent with its known epidemiology [12]. This finding underscores the need for targeted surveillance and preventive strategies in pediatric populations, particularly in postpandemic settings. Similar trends were reported by Yang et al. [13], who described how stringent public health measures during the pandemic significantly reduced the incidence of RSV and influenza in children. However, as these measures were lifted, an “immunity debt” phenomenon was observed, leading to out‐of‐season and resurgent outbreaks as children resumed normal activities [13].

An intriguing observation in our study was the relatively high proportion of co‐detections, with multiple respiratory viruses detected in the same sample. The most common co‐detections involved RV/EV together with AdV or RSV, which may suggest potential virus–virus interactions. Although some of these associations were previously considered unlikely [14], prior research from Brescia, Italy, reported similar co‐infection patterns in the prepandemic period, particularly involving RSV [15]. Understanding these interactions is critical, as viral co‐infections have been linked to increased disease severity and altered pathogenesis [14]. Future studies should explore these dynamics further, particularly in relation to SARS‐CoV‐2 co‐infections.

This study has some limitations. First, its retrospective design and single‐center data may limit the generalizability of our findings. Second, external factors such as vaccination rates and healthcare‐seeking behavior changes during the pandemic were not accounted for. Third, we lacked patient‐specific information on prior or concurrent SARS‐CoV‐2 infections, which could have influenced viral detection patterns. Future research should address these gaps by incorporating multicenter data and considering additional epidemiological variables.

In conclusion, our study highlights significant changes in the epidemiology of respiratory viruses other than SARS‐CoV‐2 over the past 7 years, shaped by the impact of the COVID‐19 pandemic. These findings underscore the need for ongoing surveillance and adaptable public health responses to effectively manage respiratory viral infections.

## Author Contributions

**Flora Marzia Liotti:** conceptualization, data curation, formal analysis, investigation, visualization, writing – original draft. **Simona Marchetti:** conceptualization, data curation, formal analysis, investigation, visualization, writing – original draft. **Sara D’Onghia:** investigation, visualization, writing – original draft. **Maurizio Sanguinetti:** project administration, resources, supervision, writing – review and editing. **Rosaria Santangelo:** conceptualization, formal analysis, writing – original draft, writing – review and editing. **Brunella Posteraro:** conceptualization, formal analysis, writing – original draft, writing – review and editing.

## Acknowledgements

Open access funding provided by BIBLIOSAN.

## Ethics Statement

The study did not require approval from the Lazio Area 3 Territorial Ethics Committee because it is an observational analysis on clinical microbiology laboratory data.

## Conflicts of Interest

The authors declare no conflicts of interest.

### Peer Review

The peer review history for this article is available at https://www.webofscience.com/api/gateway/wos/peer‐review/10.1111/irv.70079.

## Data Availability

The data that support the findings of this study are available on request from the corresponding author. The data are not publicly available because of privacy restrictions.
